# Simulation study of the effect of fasteners on the attenuation of orbital ultrasonic guided waves

**DOI:** 10.1371/journal.pone.0351111

**Published:** 2026-06-26

**Authors:** Mingqiang Wang

**Affiliations:** CHN Energy Baoshen Railway Group Co., Ltd., Ordos, China; Tongji University, CHINA

## Abstract

Ultrasonic guided wave technology offers advantages such as long-distance propagation and full-section detection for non-destructive testing of rails. However, operational rails are typically limited by fastener systems, resulting in increased guided wave energy attenuation and shorter propagation distances compared to free rails. Therefore, studying the mechanism by which fasteners influence guided wave attenuation is crucial. This paper presents a comprehensive three-dimensional solid fastener-rail contact mechanics and transient dynamics coupling model that considers the static action and complex contact relationship between fasteners and rails. The model uses an implicit-explicit combination method to simulate the propagation of guided waves in operational rails. This paper presents a simulation in which a preload force of 20 kN was applied to the fasteners, and the stress distribution of the model was analyzed. A transient analysis of the guided wave was performed under the preload force to investigate the influence of fasteners on guided wave attenuation in rails by comparing attenuation with and without fasteners. Additionally, this paper explores the impact of two contact types, penalty and motion, on wave attenuation in Abaqus/Explicit. The results demonstrate that fasteners significantly exacerbate wave attenuation in rails. The wave packet amplitude calculated using the penalty contact form is slightly greater than that calculated using the motion contact form, and this difference increases with propagation distance.

## Introduction

Ultrasonic guided wave technology has significant advantages in non-destructive testing of rails due to its long-distance propagation and full-section detection capabilities. The use of guided waves for defect detection in rails has already been applied to a certain extent [1–5]. However, when using ultrasonic guided waves for long-distance testing of operational rails, the propagation distance is often much shorter than the propagation distance in free rails [6]. This is mainly due to the fact that the rails installed on site are constrained by a large number of fastener systems. The complex stresses generated by these fasteners and the contact between the rails and structures such as rubber pads cause attenuation of the guided wave energy [7]. Therefore, to realize the practical application of ultrasonic guided waves for long-distance damage detection of operational rails, it is important to study how fasteners influence the attenuation of guided waves in rails.

Ultrasonic guided waves experience dispersion attenuation in waveguide structures due to their inherent properties. To address this issue, Wilcox et al. [8] developed a computational method to model the propagation of ultrasonic guided wave packets and examined how dispersion attenuation affects long-range detection. Legg et al. [9] proposed a frequency-domain dispersion compensation algorithm to address dispersion attenuation in ultrasonic guided waves during the long-distance detection of cable damage. This algorithm achieved detection within a 130m range. Through experimental research, Zhang et al. [10] found that the attenuation of ultrasonic guided waves in long-distance steel rails is approximately 0.4 dB/m. They reduced the attenuation by improving the guided wave transducer design. Simonetti et al. [11] excited the torsional mode of a cylindrical waveguide using a piezoelectric transducer and measured the guided wave’s phase velocity and attenuation spectrum using laser scanning.

As the connecting component between rails and sleepers, fasteners significantly alter the boundary conditions of the rails due to their stiffness and damping characteristics, thereby affecting wave propagation. Sheng et al. [12] studied the effect of vertical fastener stiffness on rail vibration energy distribution using the Timoshenko beam model and the spectral element method. They found that decreasing fastener stiffness significantly increases sound power levels in the low-frequency band, which is related to guided wave attenuation characteristics in this band. Ramatlo et al. [13] pointed out that fasteners introduce additional damping and may cause guided wave energy scattering by changing the vibration mode of the rail. The attenuation effect is more obvious in the high-frequency band. Some scholars are also concerned about the effect of fastener parameters, such as stiffness, on wave attenuation. Zhang et al. [14] used three-dimensional finite element models and experimental verification to determine that changes in fastener stiffness significantly alter the modal frequency and propagation velocity of rail vibrations. Attenuation of the vertical mode is minimal in the 1800–3600 Hz frequency band, whereas attenuation of the transverse mode peaks around 3800 Hz. Matsuoka et al. [15] quantified the effect of fastener damping on rail vibration attenuation through multi-point hammering experiments and reciprocity theorems. They found that increased damping causes guided wave energy to dissipate more quickly during propagation. The attenuation coefficient increases significantly during long-distance propagation. Carrascal et al. [16] compared the effects of different types of fasteners on guided wave energy transmission through impact attenuation experiments. They found that fastener materials and geometric designs have a greater impact on attenuation in the high-frequency range. Numerical simulation methods are widely used to thoroughly study the interaction between fasteners and rails. Yang et al. [17] used spring simulation fasteners to establish a periodic rail support model and studied the attenuation characteristics of ultrasonic guided waves in rails due to fasteners. Ryue et al. [18] established a continuously distributed rubber pad at the bottom of the steel rail and proved through simulation that the rubber pad would worsen the attenuation of guided waves in the rail base area. Fasteners generate stress in the steel rail, affecting the attenuation characteristics of ultrasonic guided waves. Some scholars have researched the effect of stress on guided wave attenuation. Mazzotti et al. [19] used a three-dimensional pre-stressed viscoelastic waveguide structure semi-analytical finite element method to study the effects of pre-stress on the group velocity, phase velocity, and attenuation characteristics of ultrasonic guided waves in rails. Liu et al. [20] experimentally investigated the effect of prestressing on the propagation behavior of ultrasonic guided waves in multiwire structures, such as steel wires. They found that increasing tensile stress reduces the energy attenuation of ultrasonic guided waves in steel wires.

Many scholars have conducted extensive research on how fasteners influence the ultrasonic guided wave attenuation characteristics of rails. However, these studies typically only consider the effects of fasteners on rails in terms of stiffness, stress, or damping. Some studies have used components, such as rubber pads, in simulation models to replace fastener systems and study wave attenuation. However, this simplified approach cannot realistically simulate the complex contact relationship and interaction between operational rails and fasteners. Thus, it cannot fully reveal the mechanism by which fasteners impact wave attenuation in rails. Abaqus is a finite element analysis software commonly used for guided wave simulation. There are usually two types of contact forms for the different components of a model: penalty contact and motion contact. However, few people have explored how these two contact forms affect guided wave simulation results. Based on this, this paper innovatively establishes a three-dimensional solid fastener-rail model to simulate the propagation of ultrasonic guided waves in rails under complex contact relationships and irregular stress distribution under the action of fastening force. It investigates the influence of fasteners on guide wave attenuation and the impact of contact forms on guide wave simulation results.

## The establishment of the fastener-rail model

### Model establishment

The transient dynamic finite element model can be used to simulate the propagation characteristics of ultrasonic guided waves in rails, thereby studying the attenuation law of guided waves under the action of the fastener system. This paper establishes a coupled solid model of contact mechanics and transient dynamics, which comprehensively considers the static interaction and complex contact relationship between the fastener and the rail. There are two main difficulties in establishing this model: First, it is challenging to simulate the propagation of guided waves in the rail under the pressure of the fastener. Second, it is necessary to simulate the complex contact interactions among various fastener components and between the fastener and the rail.

**Fig 1:**
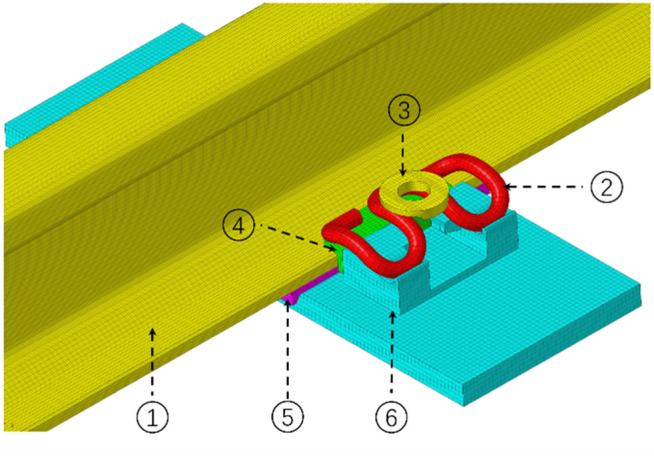
Fastener-rail model.

The fastener-rail solid finite element model established in this paper can be divided into two parts: the fastener system and the rail. The specific components are shown in Fig 1. The rail is a 60 kg/m standard rail with an elastic modulus of 210 GPa, a Poisson’s ratio of 0.3, and a material density of 7850 kg/m³. The fastener system consists of clips, bolt washers, gauge blocks, rail pads, and iron base plates. The material parameters of each component are detailed in Table 1.

**Table 1:** Fastener-rail model material parameters.

Number	Component	Density (kg/m³)	Young’s Modulus (GPa)	Poisson’s Ratio
1	Rail	7850	210	0.3
2	Bar-spring clip	7740	206	0.3
3	Bolt washer	7850	200	0.3
4	Gauge block	7850	210	0.3
5	Rail pad	1000	3	0.47
6	Base plate	7100	210	0.3

The meshing of grid elements is a crucial factor affecting computational efficiency and accuracy. In the model established in this paper, the fastener system contains multiple small irregular components, and the rail has a special-shaped cross-section. To make the cross-sectional mesh fit the contours of each component of the model as much as possible, eight-node hexahedral elements are used to mesh the model, with the maximum side length of each element not exceeding 4 mm. The result of meshing is shown in Fig 1. The computational time step of the model is 1e-6s, which ensures the accurate calculation of the wave effect.

**Fig 2:**
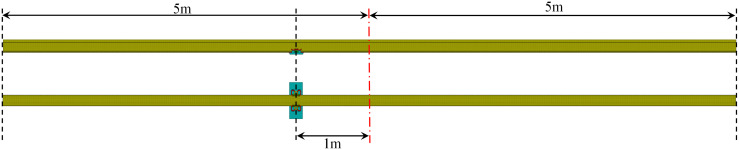
The fastener-rail integrated model.

After the model meshing is completed, the overall model assembly is carried out, as shown in Fig 2. The length of the rail is 10m. Fasteners are arranged at a position 1m away from the midpoint on one side of the rail, and no fasteners are set on the other side.

### Excitation and reception setup

**Fig 3:**
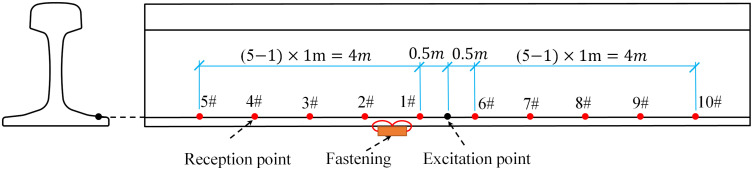
Excitation and reception setup.

Fastener systems are typically arranged at the bottom of the rail, and the stress generated by their clamping force in the rail is mainly distributed in the rail bottom area. Therefore, this paper only investigates the influence of fasteners on the guided wave attenuation in the rail bottom area. Fig 3 shows the arrangement of guided wave signal excitation and reception points. To facilitate comparative analysis of the influence of fasteners on guided waves, the excitation point is arranged at the longitudinal midpoint of the rail. Considering the feasibility of actual sensor installation, the excitation point is located on the upper surface of the rail bottom. After the guided wave signal is input, it propagates equivalently in both directions: one side includes fasteners, and the other side does not. The influence of fasteners on guided waves in the rail can be analyzed by comparing symmetric points on both sides. The signal reception points are arranged at 1m intervals, with the 1# and 6# reception points located 0.5m from the excitation point.

The ultrasonic guided wave signal is simulated by applying a transient displacement load at the excitation point. Due to the complex cross-section of the rail, numerous modes exist when guided waves propagate in the rail. The purer the excited guided wave mode, the more conducive it is to studying the influence of fasteners on guided waves in the rail, and narrowband signals can excite fewer modes. Therefore, in this simulation, a sinusoidal signal with several cycles modulated by a window function is used as the excitation signal, which has relatively concentrated energy. The commonly used Hanning window is adopted as the window function, and the formula for the excitation signal is:


$$\begin{array}{c} x(t)=\left[a_{\text{0}}-(\text{1}-a_{\text{0}})cos\frac{\text{2}\pi \cdot f_{c}\cdot t}{n}\right]\cdot sin(\text{2}\pi \cdot f_{c}\cdot t) \end{array}$$ (1)

Where: $a_{\text{0}}$ is the weighting coefficient, $f_{c}$ is the central frequency of the signal, t is time, and n is the number of cycles of the sinusoidal signal. The first half of the formula is a raised cosine window function, and when a0 = 0.5, it is called a Hanning window.

**Fig 4:**
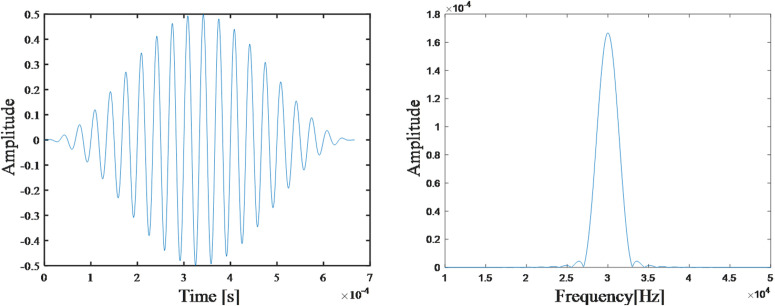
Excitation signal: (a) 30kHz Time Domain; (b) 30kHz Frequency Domain.

In this paper, the excitation signal adopted is a 20-cycle sinusoidal wave signal with a central frequency of 30 kHz modulated by a Hanning window, as shown in Fig 4.

### Simulation process

**Fig 5:**
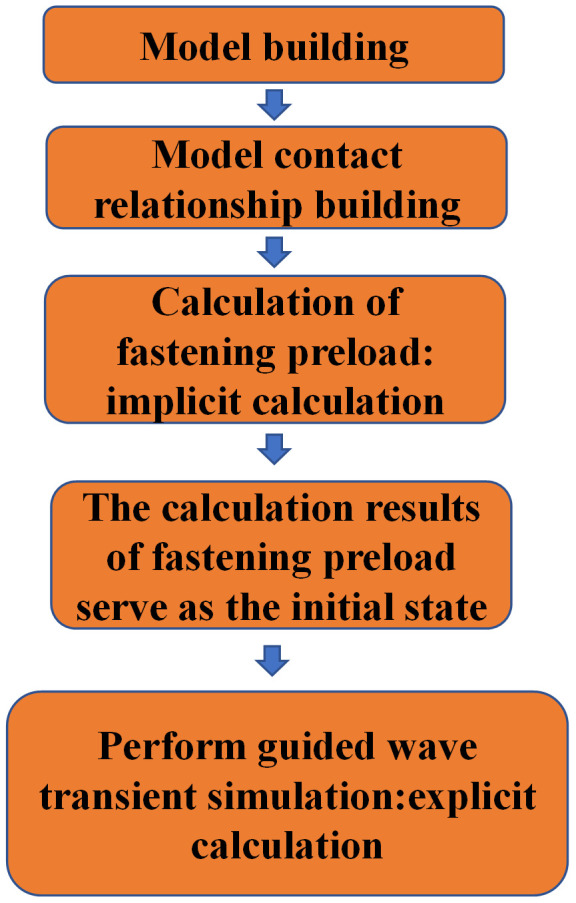
Simulation process.

As shown in Fig 5, this study uses an implicit-explicit coupling method to simulate and analyze the propagation of guided waves in rails under the action of fasteners. The specific process is as follows:

First, the implicit calculation method is used to perform static simulation calculations of the fastener preload. Based on the established finite element model with well-set contact relationships between components, a vertically downward uniform load is applied to the upper surface of the bolt washer to simulate the preloading effect of the fastener on the rail. Meanwhile, fixed constraints are applied to the bottom of the iron pad to achieve the static equilibrium of the model. Subsequently, the node displacement and stress state results obtained from the static simulation are input into the model as initial conditions for the explicit dynamic simulation, and the explicit calculation method is used to perform the transient simulation of guided waves. To simulate the actual field situation of no reflection interfaces in the rail, non-reflective boundary conditions are achieved by establishing absorption units at both ends of the rail.

## Influence of fastener contact mode on simulation

Based on the established fastener-rail guided wave transient simulation model, this chapter carries out an analysis of the influence of fasteners on the guided wave attenuation characteristics in different regions of the rail. According to the simulation process, the calculation of fastener preload is first performed to determine the initial state parameters. Subsequently, taking the initial state obtained from the preload balance calculation as the starting point, the excitation and reception signal methods selected in Chapter 2 are adopted to carry out the guided wave transient simulation analysis.

To quantify the attenuation of ultrasonic guided waves in the rail, the change in wave packet amplitude in this paper is converted into decibels using Equation (2). A negative calculation result of α indicates guided wave amplitude attenuation, while α positive result indicates guided wave amplitude increase.


$$\begin{array}{c} \alpha =-\text{2}\text{0}*{\text{log}}_{\text{1}\text{0}}(\frac{A_{\text{1}}}{A_{\text{2}}}) \end{array}$$ (2)

Where: $A_{\text{1}}$ is the amplitude of the wave packet after attenuation, $A_{\text{2}}$ is the initial amplitude of the wave packet propagation, and α is the attenuation of the guided wave amplitude.

### Simulation analysis of fastener preload

**Fig 6:**
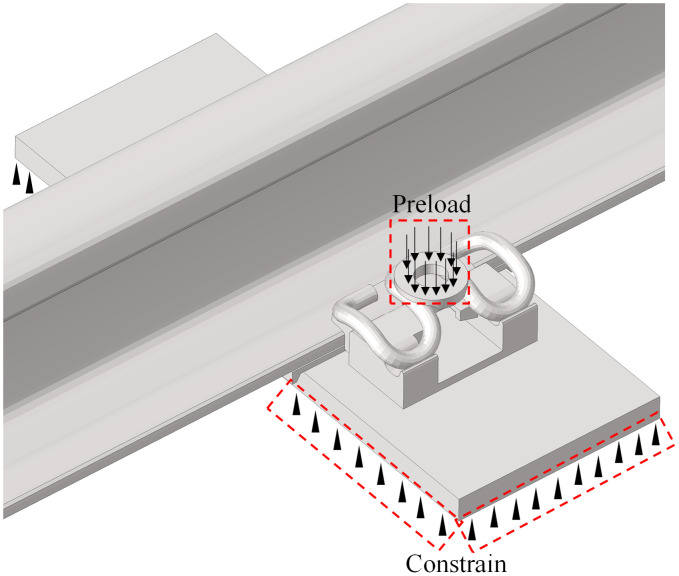
Application of preload.

In this section, the implicit calculation method is used to simulate the fastener preload. The way of applying the preload and constraining the model is shown in Fig 6. Considering the meshing characteristics of the fastener-rail model established in this paper, a fastener preload of 10kN is applied to the model.

**Fig 7:**
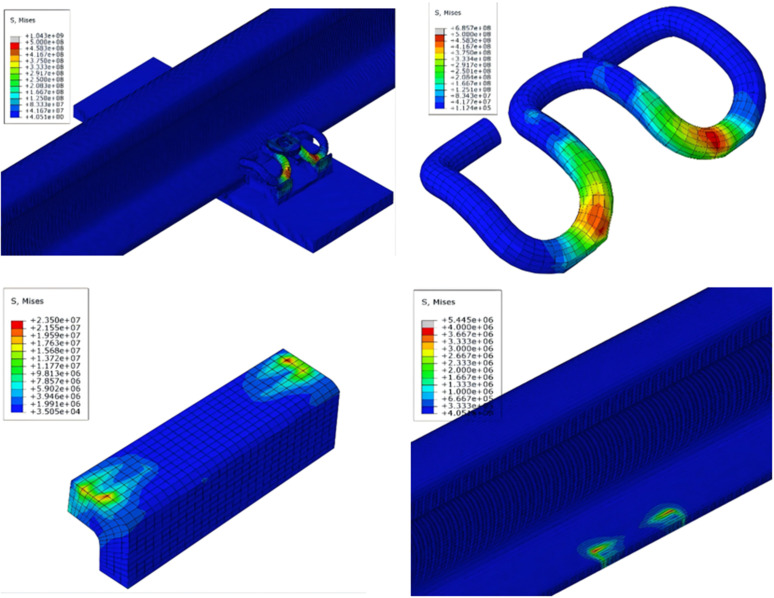
Stress cloud map of each component: (a) Overall model stress; (b) Clip stress; (c) Gauge block stress; (b) Rail stress.

Fig 7 shows the stress distribution nephogram results of the overall model, the clip, the gauge block, and the rail under the action of a 10kN preload. From the perspective of the overall model, the maximum stress occurs at the contact position between the curved section of the rear limb of the clip and the iron base plate. This position is consistent with the location of clip cracks found in the field investigation in Reference [15], and the overall maximum stress at this time is 1043MPa. For the clip itself, the maximum stress is 685.7MPa. The stress of the gauge block is mainly distributed in the contact areas with the clip and the rail. The maximum equivalent stress, which is 23.5MPa, appears in the contact area with the clip. The stress of the rail is transmitted through the gauge block, and its maximum equivalent stress is 5.45MPa. This stress occurs directly below the contact area between the clip and the gauge block, and the stress is mainly concentrated in the area of the upper surface of the rail base directly below the clip.

### Contact form

The simulation in this paper is carried out based on the commercial software Abaqus. The implicit method is used for the calculation of the clamping force, while the dynamic explicit method is employed for the calculation of guided wave propagation. In the Abaqus/Explicit dynamic explicit calculation, there are two methods for dealing with surface-to-surface contact: penalty contact and kinematic contact. These two contact methods differ in principle and application scenarios. Therefore, to investigate the influence of different contact forms on the research results, this section uses these two contact methods respectively for simulation analysis.

Penalty contact is based on the penalty function method, which simulates contact forces by introducing virtual springs at the contact interface [16]. As shown in Fig 10(a), when a penetration amount x occurs between two contact surfaces, the spring force is calculated using Equation (3). This method requires setting a certain initial penetration amount for the contact interface during model establishment, which increases the difficulty of model setup. However, it offers advantages such as high computational efficiency, suitability for large deformation problems, and good convergence. At the same time, it has the drawback of penetration errors in high-speed impact or complex contact model calculations, leading to insufficient calculation accuracy.


$$\begin{array}{c} F=k*x \end{array}$$ (3)

**Fig 8:**
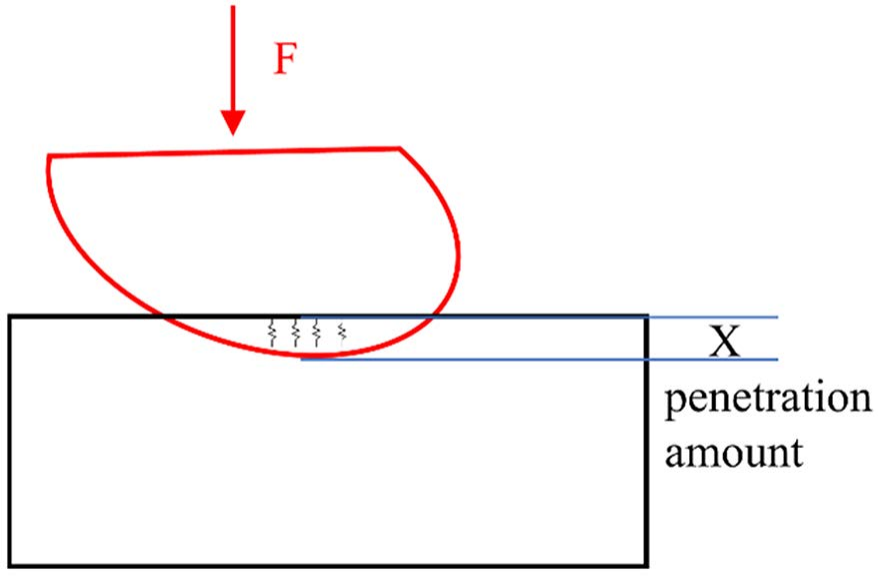
Schematic diagram of penalty contact.

Where: k is the contact stiffness, and x is the penetration amount (Fig 8).

**Fig 9:**
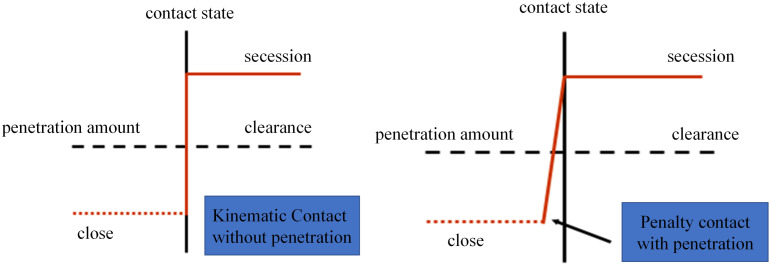
Comparison of contact methods.

Kinematic contact is based on a predictor-corrector algorithm. This method handles contact problems through a two-step iteration: in the prediction phase, the displacements of nodes without contact constraints are calculated; in the correction phase, reverse accelerations are applied to penetrating nodes to eliminate penetration and recalculate contact forces. Kinematic contact has the advantages of strictly satisfying contact constraints, having no penetration errors, and high calculation accuracy, but it has high computational costs and requires more iterations during calculation. Fig 9 shows a comparison of the two contact methods.

### Analysis of simulation results with different contact methods

**Fig 10:**
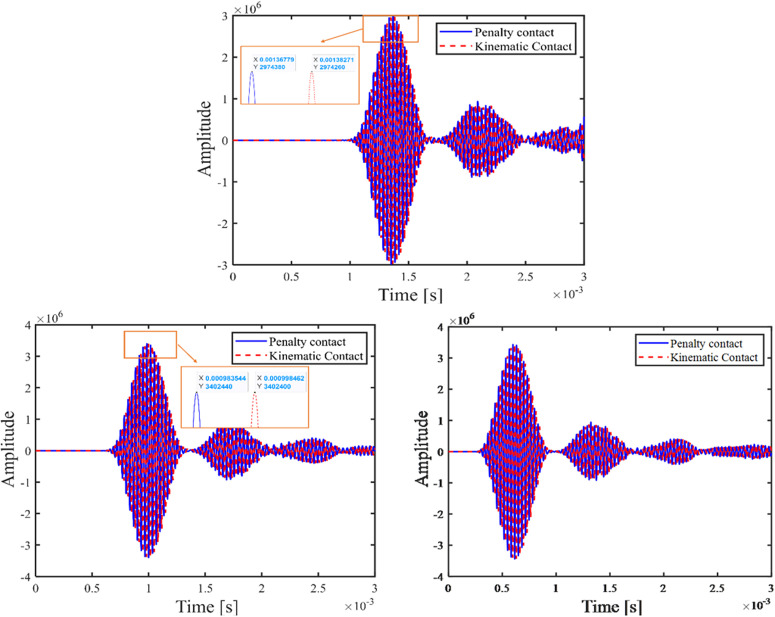
Wave packet comparison of different contact methods (a) Point No. 6.; (b) Point No. 7; (c) Point No.8.

In this section, the bolt preload calculation results from Section 3.1 are adopted as the initial state, and wave propagation simulations are conducted using the two contact methods respectively. First, a comparative analysis is performed on the signals in the undisturbed region on the right side of the rail, where the contact methods exert no impact. Fig 10a presents the comparison of wave packet signals at the 6th receiving point, where the maximum amplitudes of the two wave packets basically overlap. Fig 10b shows the wave packet comparison at the 7th receiving point, with the maximum amplitude of the wave packet obtained by penalty contact being 0.0001 dB larger than that by kinematic contact. Fig 10c displays the wave packet comparison at the 8th receiving point, where the maximum amplitude of the wave packet from penalty contact is 0.0003 dB greater than that from kinematic contact. It can be observed that in the undisturbed region where the contact methods have no influence, the amplitude difference between the wave packets under the two working conditions is extremely small and can be basically neglected.

**Fig 11:**
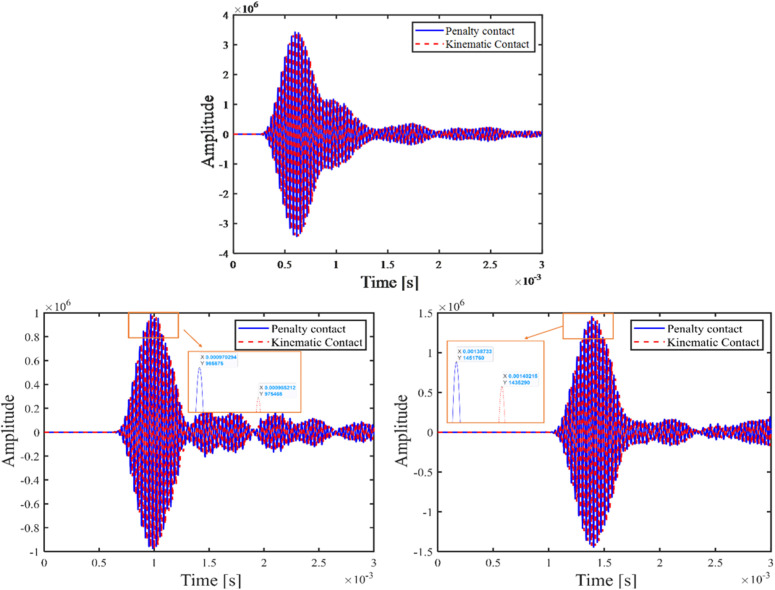
Wave packet comparison of different contact methods (a) Point No. 1; (b) Point No. 2; (c) Point No.3.

Subsequently, a comparative analysis is conducted on the region affected by the fasteners. Fig 11a shows the wave packet comparison at the signal receiving point No. 1. It can be seen that the wave packet amplitude calculated by penalty contact is basically consistent with that by kinematic contact before passing through the fastener. Fig 11b displays the wave packet comparison at the signal receiving point No. 2, where the guided wave signal has passed through the fastener. After passing through the fastener, there is a certain difference in the wave packet amplitude calculated by penalty contact and kinematic contact. The maximum wave packet amplitude of penalty contact calculated by Equation (2) is 0.09 dB larger than that of kinematic contact. Fig 11c presents the wave packet comparison at the signal receiving point No. 3, where the maximum wave packet amplitude of penalty contact is 0.10 dB larger than that of kinematic contact.

Therefore, the wave packet amplitude obtained by the penalty contact calculation is slightly larger than that by the kinematic contact calculation, and the difference between the two increases with the increase of the guided wave propagation distance. To improve the calculation accuracy, kinematic contact will be used for subsequent simulations.

**Fig 12:**
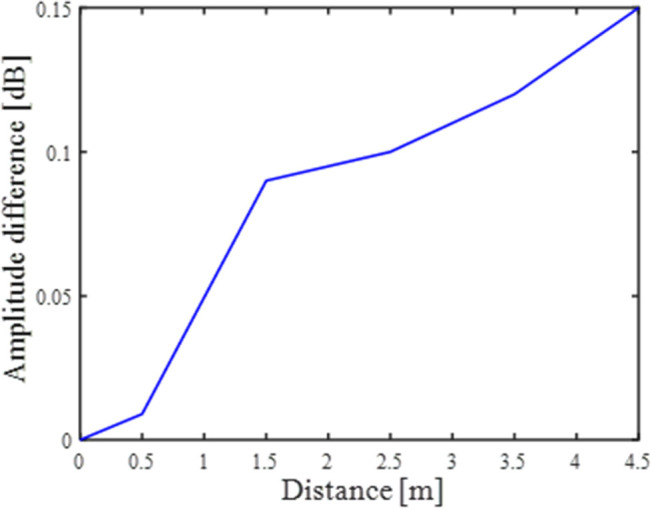
The relationship between the amplitude difference and the distance.

To better illustrate the relationship between the wave packet amplitude difference between penalty contact and kinematic contact and the propagation distance, the calculation results of each receiving point are plotted in Fig 12. The error induced by penalty contact shows an increasing trend with the growth of propagation distance. When the guided wave propagation distance exceeds 5m, this distance is measured from the excitation point and the wave travels more than 3m after passing through the fasteners, the amplitude error of the wave packet between penalty contact and kinematic contact will exceed 0.1dB.

If the research has high requirements for guided wave amplitude accuracy and the allowable error must be ≤ 0.1dB, it is recommended to stop using the penalty contact method and switch to the kinematic contact method when the guided wave propagation distance exceeds 5m. If the research only needs to analyze the attenuation trend of the guided wave and the allowable error is ≤ 1dB, the penalty contact method can be applied for the analysis of guided wave propagation distances within 10m.

## Influence of fasteners on guided wave attenuation in rails

**Fig 13:**
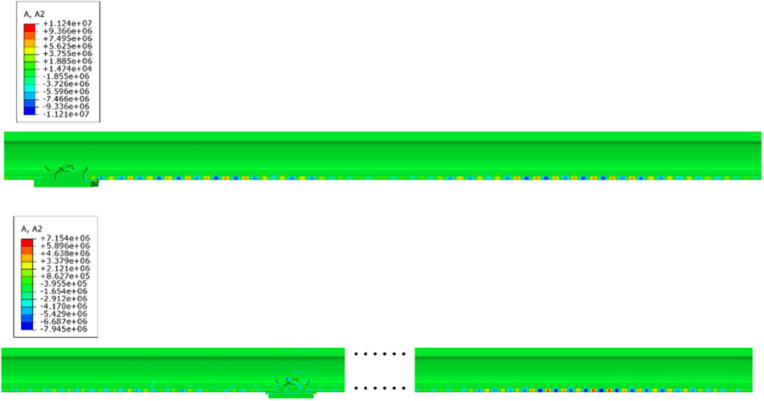
Acceleration cloud map: (a) Cloud map before passing through fastener; (b) Cloud map after passing through fastener.

Building upon the node displacements and stress states calculated under the preload in Section 3.1 as initial conditions, this section employs the explicit method with kinematic contact at the fastener-rail interface. An ultrasonic guided wave is excited at the midpoint of the rail, propagating equivalently in both directions. Fig 13 depicts the acceleration nephograms of the guided wave propagation in the rail. Fig 13a shows the nephogram before the left-side wave reaches the fastener, where the signal intensities on both sides are approximately identical. Fig 13b illustrates the nephogram after the wave passes through the fastener, revealing that the signal intensity on the unfastened side is significantly higher than that on the fastened side. Evidently, the fastener notably exacerbates the attenuation of the guided wave signal.

**Fig 14:**
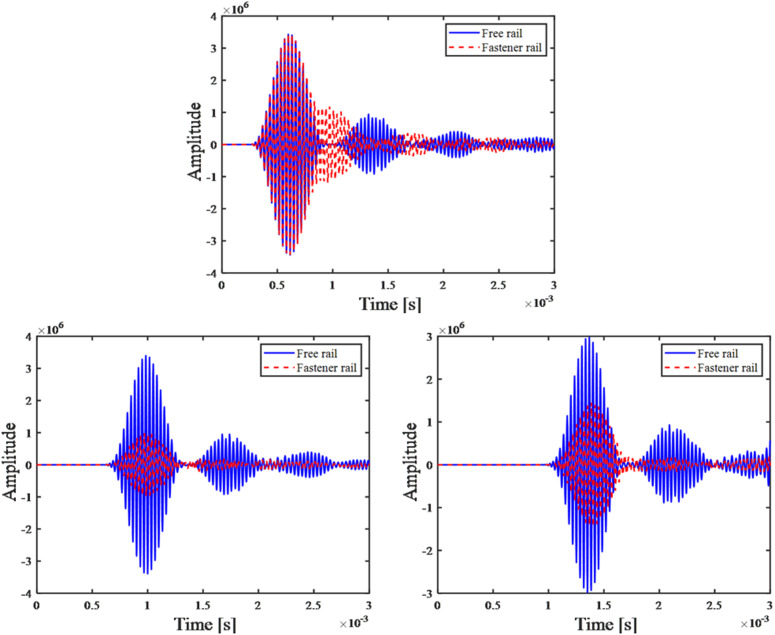
Guided wave signal comparison: (a) 1#vs6#; (b) 2#vs7#; (c) 3#vs8#.

To analyze the influence of fasteners on the attenuation of guided waves in rails in detail, the guided wave signals at each receiving point are extracted and compared. Fig 14 shows the wave packet comparison between the left and right sides of the rail. The guided wave on the fastened side is referred to as the “fastened rail,” while that on the unfastened side is called the “free rail.”

Fig 14a compares the wave packet signals at receiving points No. 1 and No. 6 in Fig 3. Since these two points are equidistant from the excitation source, they receive the signal wave packets simultaneously. By superimposing the signals, it is observed that there are certain differences between the waveforms of the free rail and the fastened rail. The different guided wave modes in the free rail have separated, while mode superposition still exists in the fastened rail. However, the amplitudes of the main wave packets are basically the same. Calculations using Equation (2) show that the wave packet amplitude of the free rail is 0.009 dB higher than that of the fastened rail. Fig 14b presents the comparison at points No. 2 and No. 7. After passing through the fastener, the wave packet amplitude on the fastened side is significantly lower than that on the free side. The free rail wave packet amplitude is 10.752 dB higher than that of the fastened rail according to Equation (2). Fig 14c shows the superimposed comparison at points No. 3 and No. 8. Here, the amplitude difference decreases to 6.23 dB. This reduction compared to points No. 2 and No. 7 is due to the exponential nature of the attenuation curve, where the decay rate slows as the amplitude decreases after the initial sharp attenuation caused by the fastener.

## Conclusions

By establishing a three-dimensional solid fastener-rail model and combining the implicit-explicit simulation method, this study breaks through the limitation of traditional research that simplifies fasteners into stiffness or damping elements. It more truly restores the guided wave propagation environment under actual working conditions, systematically investigates the propagation characteristics and attenuation mechanism of ultrasonic guided waves in rails under the action of fastener preload, and analyzes the influence of different contact forms on guided wave attenuation. The main conclusions are as follows:

(1) Under a preload of 10 kN, the maximum stress in the model occurs at the contact position between the curved section of the clip’s rear limb and the iron base plate, reaching 1043 MPa, which is consistent with the crack location of field clips. The maximum stress in the clip is 685.7 MPa, the maximum stress in the gauge block is 23.5 MPa, and the maximum stress in the rail is 5.45 MPa, concentrated in the area directly below the clip on the upper surface of the rail base. The results indicate that the fastener transfers stress through the contact interface, significantly altering the local stress state of the rail.
(2) The non-uniform influence law of fasteners on guided wave attenuation is revealed..After guided waves pass through fasteners, the wave packet amplitude on the fastened side is significantly lower than that on the unfastened side. At the No. 2/7 measuring points 1.5 meters away from the excitation point, the wave packet amplitude of the free rail is 10.752 dB higher than that of the fastened rail. Due to the exponential nature of the attenuation curve—where the decay rate slows as the wave packet amplitude decreases—the amplitude difference decreases to 6.23 dB at the No. 3/8 measuring points 2.5 meters from the excitation point. These results indicate that fasteners cause rapid dissipation of guided wave energy in rails through stress transfer and complex contact interactions, with the most pronounced attenuation effect occurring in the area near the fasteners.
(3) Different contact forms have an impact on the accuracy of guided wave simulation. Simulation results of penalty contact and kinematic contact show that the wave packet amplitudes of the two methods are basically the same at Point 1 before passing through the fastener. After passing through the fastener, the wave packet amplitude calculated by penalty contact is slightly larger than that by kinematic contact, with a difference of 0.09 dB at Point 2 and 0.10 dB at Point 3. Therefore, the difference in wave packet amplitude gradually increases with the propagation distance. This is because penalty contact has penetration errors, while kinematic contact strictly satisfies contact constraints through multiple iterations, resulting in higher calculation accuracy but also higher computational cost.

The three-dimensional solid model established in this study breaks through the traditional research method of simplifying fasteners into stiffness or damping elements. It can better fit the actual working conditions, as it can not only simulate the stiffness effect but also the influence of fastener preload and the direct contact relationship between fasteners and the rail on guided waves, greatly improving the accuracy of guided wave attenuation simulation in rails. Based on the above findings, this study suggests prioritizing the use of the three-dimensional solid coupled model to simulate the fastener-rail interaction in simulations, avoiding the neglect of contact relationships and stress distribution by traditional simplified models. In addition, the contact form should be selected according to research requirements: if high accuracy is required with an allowable error of ≤0.1dB, kinematic contact is recommended when the propagation distance exceeds 5m; if only the attenuation trend needs to be analyzed with an allowable error of ≤1dB, penalty contact can be applied for propagation distance analysis within 10m.
