# Supplementary material for: Simulation study of the effect of fasteners on the attenuation of orbital ultrasonic guided waves
Source: PLoS One. 2026 Jun 26;21(6):e0351111. doi: 10.1371/journal.pone.0351111 (PMC13308769; doi:10.1371/journal.pone.0351111)
Supplement: Attachment — Submitted filename: Detailed Response to Reviewers.docx [file pone.0351111.s001.docx]

***We thank the reviewers for their comments on our manuscript, all of which are valuable and helpful in revising and improving our work, as well as providing important guidance for our research. We have carefully considered the comments and have made corrections that we hope will meet with approval. The revised parts are marked in red in the revised manuscript.***

***The main corrections in the paper and the responses to the reviewers' comments are as follows:***

**Reviewer 1：**

Reviewer #1: The presented manuscript examines the numerical analysis of the attenuation of orbital ultrasonic guided waves caused by fasteners. This topic is of high interest since accurately characterizing the damage state of railway structures is crucial for safe operation. Therefore, the numerical simulation is enhanced by a two-step procedure. First, the fastening preload is applied to the model before the wave propagation is simulated in a second step. The numerical simulations reveal interesting results. However, some major concerns should be addressed before publication:

1) Most important is the maximum stress generated during preload application. With values of 3188 MPa and 2098 MPa, this is significantly higher than the ultimate tensile strength of steel. Is a linear simulation in this context valid? Furthermore, it is unclear which stress component is plotted in Figure 7. The unit is missing as well.

***Response***：***Thanks to the reviewers for pointing out the flaws in our paper and giving valuable suggestions. We fully agree with the reviewers' comments. There are errors in the presentation of the values 3188MPa and 2098MPa in the paper, which resulted from incorrect extraction of preloading force data and have been corrected. Meanwhile, we have labeled the stress component and indicated the element type in the legend of Figure 7.***

***For your convenience, we have included the revised content below. Words and sentences marked in red indicate revised parts, and unmarked words and sentences indicate unmodified parts. These revisions are also highlighted in red in the revised manuscript.***

***In Page 7*** ***:***

Figure 7 shows the stress distribution nephogram results of the overall model, the clip, the gauge block, and the rail under the action of a 10kN preload. From the perspective of the overall model, the maximum stress occurs at the contact position between the curved section of the rear limb of the clip and the iron base plate. This position is consistent with the location of clip cracks found in the field investigation in Reference [15], and the overall maximum stress at this time is 1043MPa. For the clip itself, the maximum stress is 685.7MPa. The stress of the gauge block is mainly distributed in the contact areas with the clip and the rail. The maximum equivalent stress, which is 23.5MPa, appears in the contact area with the clip. The stress of the rail is transmitted through the gauge block, and its maximum equivalent stress is 5.45MPa. This stress occurs directly below the contact area between the clip and the gauge block, and the stress is mainly concentrated in the area of the upper surface of the rail base directly below the clip.

|  |  |
| --- | --- |
| (**a**) | (**b**) |
|  |  |
| (c) | (d) |

**Figure 7.** Stress cloud map of each component: (**a**) Overall model stress; (**b**) Clip stress; (**c**) Gauge block stress; (**b**) Rail stress;

2) It is unclear to the reviewer why the influence of the different numerical methods is directly analyzed in the region of the fastener. It appears to be useful to analyze the effect in the undisturbed area first.

***Response***：***Thanks to the reviewers for pointing out the flaws in our paper and giving valuable suggestions. We fully agree with the reviewers' suggestions and have added an analysis of the undisturbed region prior to the analysis of the fastener-affected region. For your convenience, we have included the revised content below. It is also highlighted in red in the revised manuscript.***

***Words and sentences marked in red indicate revised parts, and unmarked words and sentences indicate unmodified parts.***

***In Page 8:***

In this section, the bolt preload calculation results from Section 3.1 are adopted as the initial state, and wave propagation simulations are conducted using the two contact methods respectively. First, a comparative analysis is performed on the signals in the undisturbed region on the right side of the rail, where the contact methods exert no impact. Figure 10(a) presents the comparison of wave packet signals at the 6th receiving point, where the maximum amplitudes of the two wave packets basically overlap. Figure 10(b) shows the wave packet comparison at the 7th receiving point, with the maximum amplitude of the wave packet obtained by penalty contact being 0.0001 dB larger than that by kinematic contact. Figure 10(c) displays the wave packet comparison at the 8th receiving point, where the maximum amplitude of the wave packet from penalty contact is 0.0003 dB greater than that from kinematic contact. It can be observed that in the undisturbed region where the contact methods have no influence, the amplitude difference between the wave packets under the two working conditions is extremely small and can be basically neglected.

|  | |  |
| --- | --- | --- |
| (**a**) | |  |
|  |  | |
| (b) | (c) | |

**Figure 10.** Wave Packet Comparison of Different Contact Methods (**a**) Point No. 6.; (**b**) Point No. 7; (**c**) Point No.8 ;

3) The acceleration scale is missing in Figure 11.

***Response***：***Thanks*** ***to the reviewers for their careful check and valuable suggestions. We fully agree with the reviewers' comments and have added the acceleration scale to Figure 11. Due to the addition of images during the revision, the sequence number of this figure has been changed to 13. For your convenience, we have included the revised content below. It is also highlighted in red in the revised manuscript.***

***Words and sentences marked in red indicate revised parts, and unmarked words and sentences indicate unmodified parts.***

***In Page 11:***

(**a**)

(**b**)

**Figure 13.** Acceleration cloud map: (**a**) Cloud map before passing through fastener.; (**b**) Cloud map after passing through fastener.

4) Wrong reference for Equation (4) in line 194.

***Response：Thanks to the reviewers for their careful check and valuable suggestions. We fully agree with the reviewers' comments and have corrected the citation error of Equation (4) in Line 194 to Equation (2). For your convenience, we have included the revised content below. Words and sentences marked in red indicate revised parts, and unmarked words and sentences indicate unmodified parts. These revisions are also highlighted in red in the revised manuscript.***

***In Line 194:***

…in this paper is converted into decibels using Equation (2)…

5) A_1 and A_0 in line 197 No multiplication operator between scalars (Equations 1 and 3),a0 in line 163

***Response***：***Thanks to the reviewers for their careful check and valuable suggestions. We fully agree with the reviewers' comments and have revised the notation of A₀ to A₂ in Equation (2), added the corresponding multiplication operator, and modified the equation's annotation accordingly. For your convenience, we have included the revised content below. These revisions are also highlighted in red in the revised manuscript.***

***Words and sentences marked in red indicate revised parts, and unmarked words and sentences indicate unmodified parts.***

***In Page 6:***

$$\alpha=-20*\log_{10}( \frac{A_{1}}{A_{2}})$$

(2)

Where: $A_{1}$ is the amplitude of the wave packet after attenuation, $A_{2}$ is the initial amplitude of the wave packet propagation, and α is the attenuation of the guided wave amplitude.

**Reviewer 2：**

Reviewer#2:The author did a simulation study to learn how the rail fasteners impact the ultrasonic guided wave attenuation. The conclusions are: 1) fasteners significantly altered the local stress state of the rail. 2)Fasteners significantly attenuated the guided wave. 3) accuracy of penalty contact calculation decreases with longer distance. My questions are listed below:

1) What do you consider to be the new finding in this paper?

***Response***：***Thanks to the reviewers for raising highly valuable questions and showing concern for the research innovation. The new findings of this paper are mainly reflected in the following three aspects:***

***A three-dimensional solid fastener-rail coupled model has been established to simulate the complex contact relationship between fasteners and rails as well as the stress distribution of fastener preload. This breaks through the limitation of traditional research that simplifies fasteners into stiffness or damping elements, and more truly restores the guided wave propagation environment under actual working conditions.***

***The non-uniform influence law of fasteners on guided wave attenuation has been revealed: significant amplitude attenuation occurs when guided waves pass through fasteners.***

***The influence of different contact forms on the accuracy of guided wave simulation has been clarified: penalty contact and kinematic contact show good consistency in results when guided waves have not passed through fasteners, but after the guided waves pass through fasteners, the wave packet amplitude of penalty contact is slightly larger than that of kinematic contact. This provides a quantitative reference for the selection of contact methods in similar subsequent simulations.***

***Corresponding explanatory content has also been added in the paper. For your convenience, the revised content is provided below. Words and sentences marked in red indicate revised parts, while unmarked words and sentences indicate unmodified parts.***

***In Page 13:***

By establishing a three-dimensional solid fastener-rail model and combining the implicit-explicit simulation method, this study breaks through the limitation of traditional research that simplifies fasteners into stiffness or damping elements. It more truly restores the guided wave propagation environment under actual working conditions, systematically investigates the propagation characteristics and attenuation mechanism of ultrasonic guided waves in rails under the action of fastener preload, and analyzes the influence of different contact forms on guided wave attenuation. The main conclusions are as follows:

…

(2) The non-uniform influence law of fasteners on guided wave attenuation is revealed..After guided waves pass through fasteners, the wave packet amplitude on the fastened side is significantly lower than that on the unfastened side. At the No. 2/7 measuring points 1.5 meters away from the excitation point, the wave packet amplitude of the free rail is 10.752 dB higher than that of the fastened rail. Due to the exponential nature of the attenuation curve—where the decay rate slows as the wave packet amplitude decreases—the amplitude difference decreases to 6.23 dB at the No. 3/8 measuring points 2.5 meters from the excitation point. These results indicate that fasteners cause rapid dissipation of guided wave energy in rails through stress transfer and complex contact interactions, with the most pronounced attenuation effect occurring in the area near the fasteners.

(3) Different contact forms have an impact on the accuracy of guided wave simulation. Simulation results of penalty contact and kinematic contact show that the wave packet amplitudes of the two methods are basically the same at Point 1 before passing through the fastener. After passing through the fastener, the wave packet amplitude calculated by penalty contact is slightly larger than that by kinematic contact, with a difference of 0.09 dB at Point 2 and 0.10 dB at Point 3. Therefore, the difference in wave packet amplitude gradually increases with the propagation distance. This is because penalty contact has penetration errors, while kinematic contact strictly satisfies contact constraints through multiple iterations, resulting in higher calculation accuracy but also higher computational cost.

…

2) Based on your simulation study, what is your recommendation for investigating the propagation of guided waves in rails? With the new, more accurate simulation method, how much did the accuracy improve compared to the traditional approach that simplifies the fasteners as either stiffness or damping.

***Response：Thanks to the reviewers for raising such valuable questions. We will supplement the suggestions for researching guided wave propagation on rails and the comparison with traditional simplified simulation methods in the paper to improve the content. Meanwhile, we also provide a detailed explanation to this question here. Based on the simulation research of this paper, we put forward the following suggestions for studying guided wave propagation on rails: prioritize the use of the three-dimensional solid coupled model to simulate the fastener-rail interaction in simulations, avoiding the neglect of contact relationships and stress distribution by traditional simplified models; and select the contact form according to research requirements.***

***Compared with the traditional method that simplifies fasteners into stiffness or damping elements, the solid fastener-rail model established by the simulation method in this paper can better fit the actual working conditions. It can not only simulate the stiffness effect but also the influence of fastener preload and the direct contact relationship between fasteners and rails on guided waves, greatly improving the accuracy. The traditional method of simplifying fasteners into stiffness usually has no effect on the attenuation of ultrasonic guided waves. In contrast, the accuracy of this paper is improved by approximately 60%.***

***For your convenience, the revised content is provided below. Words and sentences marked in red indicate revised parts, while unmarked words and sentences indicate unmodified parts.***

***In Page 14:***

…

The three-dimensional solid model established in this study breaks through the traditional research method of simplifying fasteners into stiffness or damping elements. It can better fit the actual working conditions, as it can not only simulate the stiffness effect but also the influence of fastener preload and the direct contact relationship between fasteners and the rail on guided waves, greatly improving the accuracy of guided wave attenuation simulation in rails. Based on the above findings, this study suggests prioritizing the use of the three-dimensional solid coupled model to simulate the fastener-rail interaction in simulations, avoiding the neglect of contact relationships and stress distribution by traditional simplified models. In addition, the contact form should be selected according to research requirements: if high accuracy is required with an allowable error of ≤0.1dB, kinematic contact is recommended when the propagation distance exceeds 5m; if only the attenuation trend needs to be analyzed with an allowable error of ≤1dB, penalty contact can be applied for propagation distance analysis within 10m.

3) As mentioned in this paper, the accuracy of penalty contact calculations decreases with increasing distance. At what point should people stop using this method? Could you provide a plot showing the error versus distance?

***Response: Thanks to the reviewers for raising highly valuable questions. We will supplement a figure demonstrating the relationship between the calculation accuracy of penalty contact and distance in the paper, and propose indicators for determining which contact form to adopt. For your convenience, we have included the supplemented content below. Words and sentences marked in red indicate revised parts and unmarked words and sentences indicate unmodified parts.***

***In Page 10:***

To better illustrate the relationship between the wave packet amplitude difference between penalty contact and kinematic contact and the propagation distance, the calculation results of each receiving point are plotted in Figure 12. The error induced by penalty contact shows an increasing trend with the growth of propagation distance. When the guided wave propagation distance exceeds 5m, this distance is measured from the excitation point and the wave travels more than 3m after passing through the fasteners, the amplitude error of the wave packet between penalty contact and kinematic contact will exceed 0.1dB.

If the research has high requirements for guided wave amplitude accuracy and the allowable error must be ≤ 0.1dB, it is recommended to stop using the penalty contact method and switch to the kinematic contact method when the guided wave propagation distance exceeds 5m. If the research only needs to analyze the attenuation trend of the guided wave and the allowable error is ≤ 1dB, the penalty contact method can be applied for the analysis of guided wave propagation distances within 10m.

**Figure 12.** The relationship between the amplitude difference and the distance.

4) Figure 10 is hard to read, could you extend the plot to make it clearer?

***Response: Thanks to the reviewers for their careful check and valuable suggestions. We fully agree with the reviewers' comments and have enlarged the detailed parts of Figure 10. Due to the addition of images during the revision, the sequence number of this figure has been changed to 11. For your convenience, we have included the revised content below. These revisions are also highlighted in red in the revised manuscript.***

***In Page 10:***

|  | |  |
| --- | --- | --- |
| (**a**) | |  |
|  |  | |
| (b) | (c) | |

**Figure 11.** Wave Packet Comparison of Different Contact Methods (**a**) Point No. 1.; (**b**) Point No. 2; (**c**) Point No.3 ;

5) Please provide colorbar for Figure 11.

***Response：Thanks to the reviewers for their careful check and valuable suggestions. We fully agree with the reviewers' comments and have added the acceleration scale to Figure 11. Due to the addition of images during the revision, the sequence number of this figure has been changed to 13. For your convenience, we have included the revised content below. It is also highlighted in red in the revised manuscript.***

***Words and sentences marked in red indicate revised parts, and unmarked words and sentences indicate unmodified parts.***

***In Page 11:***

(**a**)

(**b**)

**Figure 13.** Acceleration cloud map: (**a**) Cloud map before passing through fastener.; (**b**) Cloud map after passing through fastener.
